# Word Skipping in Chinese Reading: The Role of High-Frequency Preview and Syntactic Felicity

**DOI:** 10.1037/xlm0000738

**Published:** 2019-06-27

**Authors:** Chuanli Zang, Hong Du, Xuejun Bai, Guoli Yan, Simon P. Liversedge

**Affiliations:** 1 Academy of Psychology and Behavior, Tianjin Normal University, and School of Psychology, University of Central Lancashire; 2 Academy of Psychology and Behavior, Tianjin Normal University; 3 School of Psychology, University of Central Lancashire

**Keywords:** eye movements, skipping, preview, syntactic felicity, Chinese reading

## Abstract

Two experiments are reported to investigate whether Chinese readers skip a high-frequency preview word without taking the syntax of the sentence context into account. In Experiment 1, we manipulated target word syntactic category, frequency, and preview using the boundary paradigm (Rayner, 1975). For high-frequency verb targets, there were identity and pseudocharacter previews alongside a low-frequency noun preview. For low-frequency verb targets, there were identity and pseudocharacter previews alongside a high-frequency noun preview. Results showed that for high-frequency targets, skipping rates were higher for identical previews compared with the syntactically infelicitous alternative low-frequency preview and pseudocharacter previews, however for low-frequency targets, skipping rates were higher for high-frequency previews (even when they were syntactically infelicitous) compared with the other 2 previews. Furthermore, readers were more likely to skip the target when they had a high-frequency, syntactically felicitous preview compared to a high-frequency, syntactically infelicitous preview. The pattern of felicity effects was statistically robust when readers launched saccades from near the target. In Experiment 2, we assessed whether display change awareness influenced the patterns of results in Experiment 1. Results showed that the overall patterns held in Experiment 2 regardless of some readers being more likely to be aware of the display change than others. These results suggest that decisions to skip a word in Chinese reading are primarily based on parafoveal word familiarity, though the syntactic felicity of a parafoveal word also exerts a robust influence for high-frequency previews.

During reading, we make saccades frequently in order to position upcoming (i.e., not yet fixated) words in a text into the center of the visual field, foveal vision, where visual acuity is highest for optimal word identification. Whereas the majority of words in an alphabetic text are fixated during first pass reading, up to 30% of them do not receive a direct fixation, but are initially skipped. Therefore, foveal processing of these words does not occur (Rayner, 1998, 2009). In these cases, word identification has to occur on the basis of preprocessing of the word when it is in the parafovea, in combination with constraint provided by the sentential context that readers have previously read and any processing of the word that may take place during fixations made after it has been skipped. Note, also, that a small proportion of skips occur due to saccadic error, and we will not consider these in the context of the present study. Issues regarding how, and to what extent, readers use parafoveal and contextual information in making skipping decisions during Chinese reading, are not well understood. These will be our focus in our experiments.

Previous research in reading of alphabetic languages has clearly demonstrated that parafoveal information has a substantial impact on the decision as to whether the upcoming word will be skipped. The most substantive visual influence on skipping of a parafoveal word is its length and the distance of the eyes from it (the so-called launch site distance): Short words are more likely to be skipped than long words (e.g., Brysbaert, Drieghe, & Vitu, 2005; Rayner, Slattery, Drieghe, & Liversedge, 2011; Vitu, O’Regan, Inhoff, & Topolski, 1995). For example, Vitu, O’Regan, Inhoff, and Topolski (1995) observed that one-letter words were skipped about 80% of the time, three-letter words were skipped about 60%, five-letter words were skipped about 30% of the time, whereas words that were seven letters or longer were skipped only 10% of the time. Moreover, the closer a preceding fixation is to a parafoveal word, the higher the probability that this word will be skipped (e.g., Drieghe, Rayner, & Pollatsek, 2005; Fitzsimmons & Drieghe, 2011; White, Rayner, & Liversedge, 2005a).

It is also well-established that when word length is matched, high-frequency words are more likely to be skipped than low-frequency words (e.g., Brysbaert et al., 2005; White, 2008), and repeated words are more likely to be skipped than nonrepeated words (Choi & Gordon, 2014; Gordon, Plummer, & Choi, 2013; Lowder, Choi, & Gordon, 2013). In comparison with the influence of word frequency (and possibly, word repetition), a much stronger language-related influence on word skipping is the effect of contextual constraint: Words that are predictable from the preceding context are skipped more frequently than words that are less predictable (e.g., Balota, Pollatsek, & Rayner, 1985; Brysbaert et al., 2005; Drieghe et al., 2005; Fitzsimmons & Drieghe, 2013; Rayner et al., 2011). These effects of frequency, predictability and launch site on skipping decisions are pervasive, and have also been shown to hold for nonalphabetic unspaced languages like Chinese (e.g., Liversedge et al., 2014; Rayner, Li, Juhasz, & Yan, 2005; Zang, Fu, Bai, Yan, & Liversedge, 2018; see Zang, Liversedge, Bai, & Yan, 2011 for a review).

The phenomenon of word skipping provides insight into the time course of lexical identification and context integration during reading. Even though current computational models of eye movements in reading such as E-Z Reader (Reichle, 2011; Reichle, Rayner, & Pollatsek, 2003; Reichle, Warren, & McConnell, 2009) and SWIFT (Engbert & Kliegl, 2011; Engbert, Nuthmann, Richter, & Kliegl, 2005; Schad & Engbert, 2012) differ on some fundamental theoretical issues (e.g., serial vs. parallel lexical processing), they assume that word length, frequency, and predictability are the critical factors that drive the eye guidance system when making decisions regarding whether to skip an upcoming word. Specifically, E-Z Reader assumes that lexical processing is serial, and this process is causative with respect to moving the eyes from one word to the next. The decision to skip a parafoveal word is triggered by a successful familiarity check of this word (i.e., completion of the first stage, *L*_1_, of the identification of the word). In contrast, the SWIFT model assumes that lexical processing occurs in parallel, and multiple words within the perceptual span are processed simultaneously. Skipping of a word is determined by the lexical activation of the adjacent parafoveal word relative to the activation levels of words to its right that fall within the perceptual span. The SWIFT model, therefore, allows for word skipping even if parafoveal processing of a word is incomplete. Both the E-Z Reader model and SWIFT model predict that skipping occurs with increased probability when a word is short, frequent or highly constrained from the preceding context, though the relative contribution of these influences on skipping is not immediately clear. Furthermore, to date, there has been only a limited amount of experimental work to determine how these factors combine to affect word skipping decisions.

Contextual constraint can exert an influence over the predictability of a word in more than one way. Context is most often thought about as semantically constraining the words that might potentially appear downstream in a sentence with the semantic meaning of those potential words determining their likelihood of appearance. For example, the meaning of the sentence fragment *the shrubs in the greenhouse were planted by the . . .* makes it more likely that the semantically relevant word *gardener* will appear downstream than the less relevant word *astronaut*. To this extent, cloze tasks in which participants are required to complete a sentence frame up to, but not including a target word, capture how contextual meaning makes some words more likely parafoveal candidates on the basis of their own semantic meaning compared with others (that differ semantically). However, the nature of the preceding sentence also provides another form of constraint over the likelihood of possible upcoming words, namely, the syntactic context within which a word appears. For example, the grammatical form of the sentence frame *the shrubs in the greenhouse were planted by the . . .* makes it certain that the following word will either be an adverb, an adjective or a noun. In this way, based on the syntactic form of the preceding sentence context, the possibility that the upcoming word is, say, a verb is ruled out. In this way, the syntactic form as well as the semantic meaning of sentence context provides a predictability constraint. Note also that both these sources of constraint exert an influence over the nature of the completions participants provide in cloze tasks.

Angele and Rayner (2013) investigated the relative contribution of syntactic context and parafoveal information on word skipping in English text reading, and examined skipping rates for the very high-frequency article *the* in syntactically legal and illegal contexts in a gaze-contingent boundary experiment (Rayner, 1975). They asked participants to read sentences with three-letter target verbs like: *She was sure that she would ace all the tests*, in which the target verb is *ace*. Before the readers’ eyes crossed an invisible boundary located to the left of the target, the preview for the target was: (a) the article *the*, which was syntactically infelicitous in relation to the preceding sentence context; (b) the syntactically correct identity; or (c) a dissimilar nonword preview. Once the eyes crossed the boundary, the preview changed to the target word and thus participants always saw the correct target word. Angele and Rayner (2013) found that participants skipped the target up to 51% of the time when the preview was the article *the*, even though it was not syntactically permissible in the target position based on preceding sentence context. In contrast, the skipping rates for the syntactically correct identical preview were much lower (only 29%). Abbott, Angele, Ahn, and Rayner (2015) further demonstrated that even when the article preview *the* appeared in a highly constrained context that strongly predicted a target verb (rather than *the*), readers still preferentially skipped the target with *the* as a preview. These results indicate that a decision to skip the definite article *the* is mainly based on parafoveal information about the upcoming word rather than the constraints of syntactic context.

In another experiment, Angele, Laishley, Rayner, and Liversedge (2014) used a similar paradigm to Angele and Rayner (2013) and showed that this skipping effect was not specific to the extremely high-frequency function word *the*, but also applied to the other short high-frequency content words. For example, in the sentence “*The increasingly dim light made it hard to see*,” readers skipped the target word (*dim*) more often when the preview for that word was a syntactically illegal, high-frequency word (*dog*) relative to the syntactically legal, low-frequency identity preview, again, demonstrating that the characteristics of the parafoveal word were more influential over skipping than was context. However, contrasting findings have been obtained by Brothers and Traxler (2016). They manipulated the syntactic validity of an upcoming word using the boundary paradigm, comparing syntactically invalid previews like *bedroom*, with syntactically valid identity previews like *suggest* in the sentence *If you visit the airport, I would suggest/bedroom arriving two hours early*. Brothers and Traxler found that readers were more likely to skip words that were syntactically felicitous with preceding context than those that were syntactically infelicitous (see also Snell, Meeter, & Grainger, 2017 for similar results).

More recently, Veldre and Andrews (2018a) conducted a further investigation to assess whether skipping effects were due to syntactic violation or contextual plausibility of a preview. In Experiment 1, they used sentences such as: *She eventually found a spare stool/glass/uncle/begin behind the crowded bar*, in which the parafoveal preview was identical to the target (*stool*); contextually plausible and of the same word class as the target (*glass*); contextually implausible and of the same word class as the target (*uncle*); or contextually implausible and of a different word class to the target (*begin*). They found that target skipping rates were higher in the identical and plausible conditions than in either of the implausible conditions, with comparable skipping rates in both implausible conditions. Based on the results from the implausible conditions it appears that skipping decisions were based on the preview’s contextual plausibility rather than its grammatical class. In Experiment 2, Veldre and Andrews (2018a) further manipulated the syntactic validity of the preview to examine whether skipping effects still occurred when previews were contextually plausible. For example, in the sentence *Her plane will probably refuel/depart/landed/stroke later than expected this afternoon*, the preview was identical to the target (*refuel*); semantically and syntactically plausible (*depart*); semantically plausible but syntactically illegal (*landed*); or semantically implausible but of the same syntactic class as the target (*stroke*). They found that skipping rates were higher in the identical and plausible conditions than in either of the latter two conditions. Overall, these findings provide evidence that under some circumstances such as when a word is semantically plausible based on sentential context, syntactic constraints on skipping do occur.

The diverging findings we have discussed above might arise due to the use of words of different length across studies. All the target words in Angele and colleagues’ experiments (Abbott et al., 2015; Angele et al., 2014; Angele & Rayner, 2013) were three letters long, which were shorter than those used in Brothers and Traxler’s (2016) study (four to seven letters long, on average five letters, see also Snell et al., 2017; Veldre & Andrews, 2018a). Shorter words are more frequent and visually familiar, and therefore more potentially identifiable in the parafovea relative to longer words. Furthermore, the short words used by Angele and colleagues (Abbott et al., 2015; Angele et al., 2014; Angele & Rayner, 2013) were very high frequency and very familiar to readers, whereas those used by Brothers and Traxler (2016) were less so. For this reason, perhaps, context exerted less of an influence over word skipping in Angele and colleagues’ studies (Abbott et al., 2015; Angele et al., 2014; Angele & Rayner, 2013) relative to that observed in the study by Brothers and Traxler (2016) and Snell et al. (2017).

At this point, it is perhaps helpful to summarize the findings we have discussed so far. To us, a critical question concerns whether a reader identifies a word before they skip it, and more particularly, whether this occurs to a similar degree based on semantic contextual constraints relative to syntactic contextual constraints. From our perspective, semantic contextual constraint represents a relatively strong influence over the likely identity of a parafoveal word (i.e., the candidate set of potential parafoveal words is small), whereas syntactic contextual constraint represents a relatively weak influence. Semantic context has the potential to guide the reader to a relatively small set of particular words with a specific meaning, whereas syntactic context can, at best, guide the reader to an entire syntactic category of words. Given this, it seems reasonable to suggest that visual information about a parafoveal word will be more discriminatory with respect to the identity of that word in the former, relative to the latter, situation. The critical point to take from this is that if the likelihood of word skipping in reading is shown to be influenced by syntactic contextual constraints, as suggested by Brothers and Traxler (2016), then it is also likely that information about the upcoming word’s identity that is derived from parafoveal vision will too have played a more significant role. Therefore it might be the case that if readers receive a preview of a higher frequency word in the parafovea, even when it is syntactically infelicitous they might be more likely to skip it compared with when the preview is of a lower frequency word. Further work may allow us to determine the extent to which the visual familiarity of parafoveal words and the influence of syntactic context jointly constrain decisions of word skipping in reading.

Written Chinese is a good candidate language in which to investigate what actually influences whether or not an upcoming word is skipped, as words in Chinese are quite short, with approximately 90% of them being one or two characters long. As a consequence, variance in word length in Chinese is much lower relative to alphabetic languages (Zang, Fu, et al., 2018; see also Li, Zang, Liversedge, & Pollatsek, 2015; Zang et al., 2011 for reviews). Characters in Chinese occupy the same unit of space but are formed from strokes and have different degrees of visual complexity. Compared with English, visual information in written Chinese is densely packed, and thus, more parafoveal information may be potentially available for readers from upcoming words before decisions are made about skipping a word (Vasilev & Angele, 2017). However, given that there are no explicit visual cues (i.e., interword spaces), or inflectional indicators (i.e., lexical categories, number, tense, etc.) to demarcate word boundaries, or mark words’ syntactic properties, the issue of how readers identify the constituent units (e.g., words) and move their eyes in relation to those units in Chinese reading is very important and such processing might be different from that which occurs in English reading.

The continuous visual uniformity of Chinese text may lead one to assume that saccadic targeting, and therefore word skipping, may arise entirely due to saccades that are made in a way that has minimal relation to the characteristics of sentential content. If this was the case, then there would be little systematicity in relation to the nature of word skipping in Chinese reading. However, this is not the case. As for alphabetic languages, word length, word frequency, and word predictability have all been shown to affect word skipping probability in Chinese (Zang et al., 2011), and the visual complexity of a word, as characterized by stroke complexity, has also been shown to have an effect (Liversedge et al., 2014). These studies demonstrate a very important point. Assuming that to skip a word in Chinese, readers must know where that word ends (or have an estimate of where it ends), then for at least a proportion of fixations they must segment the upcoming text prior to initiating a saccade to skip the next word. To be very clear, collectively, these studies demonstrate unequivocally that Chinese readers engage in word segmentation (or at least make segmentation estimates) of parafoveal text.

A further noteworthy consideration is that Chinese readers may depend upon context for the interpretation of text to a greater degree than readers of spaced alphabetic languages. Recall that Chinese is written without inflectional markers or morphological cues to aspects of meaning. For example, the Chinese word 调查 means either *investigate* or *investigation*, and only through consideration of sentential context within which it appears can a reader know whether the word is a noun or a verb. Given the lack of specificity in relation to orthographic form in Chinese, it seems reasonable to assume that there may be a greater dependence on contextual information for successful sentence interpretation.

Recently, Zang, Zhang et al. (2018) investigated skipping for high-frequency particle *de* during Chinese reading in a boundary paradigm experiment. Participants read sentences including a single-character target word with an identical preview, a syntactically illegal high-frequency particle *de* preview, or a pseudocharacter preview. Zang, Zhang et al. (2018) found that Chinese readers were more likely to skip the target word when they had a *de* preview than the other two conditions suggesting that parafoveal processing rather than syntactic context primarily affects *de* skipping in Chinese reading. This effect is entirely consistent with the findings reported by Angele and Rayner (2013) for *the* skipping in English reading.

Extending the work of Zang, Zhang et al. (2018), and further investigating the relative contributions of parafoveal processing and syntactic context in Chinese word skipping during reading, we report two experiments in the present study. In Experiment 1, we employed the boundary paradigm to further examine whether the increased *de* skipping effect is specific to the particle *de*, or it is associated with a broader range of other high-frequency words, and whether any such effects hold for words in different syntactic categories (e.g., verbs and nouns). In Experiment 2, we assessed whether these effects were due to individual differences in display change awareness. The basic experimental design was similar to the one in Angele et al. (2014) but with additional experimental control. Specifically, we manipulated target word frequency (high or low frequency) and preview with a correct syntactically felicitous identity preview, a nonsense pseudocharacter preview, or a syntactically infelicitous higher or lower frequency alternative preview. Half of the target words were nouns and the other half were verbs. For the noun targets, the alternative previews were verbs, thereby resulting in the syntactic infelicity with respect to preceding context, and vice versa. To be clear, this meant that our previews prior to the boundary change differed with respect to both lexical identity and syntactic category in relation to target. Importantly, each pair of high- or low-frequency targets were inserted into an identical sentence frame, which allowed us to directly compare eye movement measures across all conditions without any confounding in relation to the sentence context (cf., Angele et al., 2014).

Based on previous findings, we expected that the frequency of the preview and sentence context might affect the probability that readers skip the upcoming target word. If the frequency alone affected target word skipping, then a higher frequency preview should result in more skipping than a low-frequency preview. However, if the syntactic context alone affected target word skipping, then a syntactically felicitous preview will result in more skipping than a syntactically infelicitous preview. Alternatively, syntactic context and frequency may interactively influence target word skipping, and if this occurred, then effects of frequency on skipping should be modulated by syntactic context such that readers should be more likely to skip a high-frequency, syntactically felicitous preview than previews in the other conditions. Additionally, assuming that the amount of parafoveal preview obtained from the upcoming word is largely determined by launch site, we expected any skipping effects to be more pronounced when the launch site was closer to the target word on the fixation prior to a skip. In terms of fixation times, we predicted a standard preview effect on the target word such that readers would fixate the target for less time when the preview was identical than when it was dissimilar (i.e., the alternative and pseudocharacter previews). Finally, the pseudocharacter preview would likely produce increased fixation durations on the pretarget character (visual parafoveal-on-foveal effects) and this effect may spill over onto the posttarget character (as per Angele et al., 2014).

## Experiment 1

### Method

#### Participants

Seventy-two students (mean age = 22 years, *SD* = 2.5) from Tianjin Normal University, participated in the experiment. All were native Chinese speakers with normal or corrected-to-normal vision, and were naive to the purpose of the experiment.

#### Apparatus

Participants’ eye movements were recorded with an SR Research EyeLink1000 plus eye tracker (sampling rate = 1000 Hz). Sentences were displayed on a 17-in. SAMSUNG SyncMaster 959NF monitor with a refresh rate of 120 Hz. The stimuli were presented in Song font in black on a white background. Viewing was binocular while only the right eye was recorded. Viewing distance was approximately 74 cm, and each Chinese character subtended approximately 1.1° of visual angle.

#### Materials and design

We selected 120 pairs of single high- and low-frequency target words in the Cai and Brysbaert (2010) corpus. Half of the target words were nouns and the other half were verbs. The high-frequency words had a mean word frequency of 194 per million (range = 30–1,300, *SD* = 253), while the low-frequency words had a mean word frequency of 2 per million (range = 0.06–6, *SD* = 2). The difference in frequency was significant, *t*(119) = 8.3, *p* < .001. To confirm that the Chinese readers would agree on the word class of these target words, we required 20 participants who did not participate in the eye tracking experiments to indicate the word class for each target word. The mean reliability produced 95% agreement (*SD* = 15%) for high-frequency targets and 92% agreement (*SD* = 15%) for low-frequency targets, and there were no differences between the two, *t*(19) = 1.60, *p* > .05. We constructed 120 sentence frames, with each pair of words inserted into the middle of a sentence frame. All sentences were between 16 and 21 characters in length (*M* = 18 characters), and were tested for naturalness and predictability. For the naturalness norms, 50 participants (25 in each of the high- and low-frequency word condition) who did not take part in the eye-tracking experiment, were required to rate sentence naturalness on a 5-point scale (1 = *very unnatural*, 5 = *very natural*). The mean naturalness score was 4.0, and there was no difference between the high- and low- frequency conditions, *t*(119) = 1.29, *p* > .05. For the predictability norms, a separate group of 25 participants were required to perform a sentence-completion task, in which they were given 120 sentence frames up to the target words and were asked to provide the following words to complete each sentence. Participants produced the target words less than 1.6% of the time (*SD* = 6%), indicating that all target words were unpredictable from the prior sentence contexts, *t*(119) = 1.58, *p* > .05.

**Figure 1 fig1:**
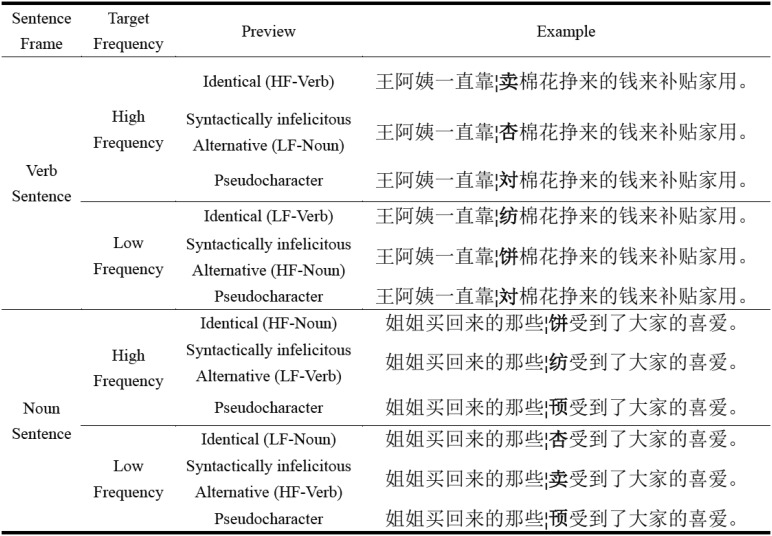
An example set of sentences from the present study. The vertical black line represents the position of the invisible boundary. When readers’ eyes crossed the boundary, the preview changed to the target word (the target and preview words are in bold). English translation for the verb sentence version is “Aunty Wang has been relying on **selling/spinning** cotton for money to support her family,” and for the noun sentence version is “My sister bought the **cakes/apricots** that were popular with all of us.”

The gaze-contingent boundary paradigm (Rayner, 1975) was used to manipulate the preview of the target word. There were three preview conditions: a correct preview that was identical to the target word, a syntactically infelicitous higher or lower frequency alternative preview, or an unrelated character that did not share any radicals with the target word and was visually, phonologically, and semantically dissimilar to its corresponding target word. Furthermore, we ensured that these characters were extremely low frequency and appeared very, very rarely in the Chinese language. In a prescreen test involving 10 participants, none were recognized as real Chinese characters (i.e., participants categorized them as pseudocharacters). More specifically, for high-frequency verb targets, there were identity and pseudocharacter previews alongside a low-frequency noun preview. For low-frequency verb targets, there were identity and pseudocharacter previews alongside a high-frequency noun preview. Presenting a noun preview for the target verb, and presenting a verb preview for a noun target, resulted in a syntactic violation. The number of strokes was counterbalanced across conditions (high-frequency word stroke number: *M* = 8.7, *SD* = 2.6; low-frequency word stroke number: *M* = 9.0, *SD* = 2.6; pseudocharacter stroke number: *M* = 8.9, *SD* = 2.6; *F* < 1.88, *p* > .05). An example set of sentences under the different conditions is shown in Figure 1.[Fig-anchor fig1]

The experiment was a 2 (Target Word Frequency: High-Frequency or Low-Frequency) × 3 (Preview: Identical, Syntactically Infelicitous Lower/Higher Frequency Alternative, or Pseudocharacter Preview). Six files were constructed, with each file containing 120 experimental sentences (20 sentences in each condition). Conditions were rotated across files according to a Latin square design. The experimental sentences were interspersed with 20 filler sentences without any changes that appeared throughout each file. Both experimental and filler sentences were presented randomly. In addition, eight practice sentences were presented at the beginning of the experiment. Yes/no comprehension questions followed 40 of the sentences. Participants were required to answer these correctly by making a button press response. Each participant read sentences only from one of the six files, and in total each participant read 148 sentences.

#### Procedure

Participants were instructed to read each sentence silently and carefully for comprehension. They were informed that they would occasionally be presented with comprehension questions after some of the sentences, and they were required to press a response key on the button box when they had finished reading the sentence and felt they understood it. The participant’s head was stabilized using a head- and chin-rest. At the beginning of the experiment, a three-point calibration procedure was carried out with average calibration error below 0.25 degrees of visual angle. After a successful calibration, practice sentences were presented for participants to become familiar with the procedure, then the experimental sentences were presented in turn. Following each sentence, the calibration was checked and participants were recalibrated whenever necessary. The entire experiment lasted approximately 35 min.

### Results

Participants’ comprehension accuracy was high (*M* = 94%, range = 88%–100%), indicating that they fully understood the sentences. All fixations shorter than 80 ms or longer than 1,200 ms were excluded from the analyses. We also excluded any trials for the following reasons: (a) a track loss occurred or there were fewer than three fixations in total (0.25%); (b) eye movement measures were above or below three standard deviations from each participant’s mean (0.5%); (c) a blink occurred during display changes or during a fixation on the target word, as well as trials in which the display changes occurred in an untimely or delayed manner (16.5%).

Analyses were conducted on a number of eye movement measures on the target words, as well as the pretarget and posttarget words. These measures included *skipping probability* (SP, the likelihood of a word not receiving a fixation during first pass reading), *first fixation duration* (FFD, the duration of the first fixation on a word, regardless of how many fixations the word received during first pass reading), *gaze duration* (GD, the sum of all first-pass fixations on a word before leaving it), and *go-past time* (go-past, the sum of all the fixations on a word from the first fixating the word until the reader makes a saccade to the right of the word).

We analyzed data using linear mixed-effects models (LMM) using the lme4 package (Version 1.1–13; Bates, Maechler, Bolker, & Walker, 2015) within the R Environment for Statistical Computing (R Core Team, 2014). The model included fixed effects, target word frequency, and preview condition, as well as their interaction. For preview condition, we used the successive contrasts, comparing the identical with the syntactically infelicitous alternative preview, and the syntactically infelicitous alternative preview with the pseudocharacter preview. In addition, participants and items were entered as crossed random effects. The random effects structure of the model was determined by starting with the maximal random effects structure (Barr, Levy, Scheepers, & Tily, 2013), but was further trimmed down if the maximum random model did not converge, probably due to missing values that were related to the high skipping rates. Fixation times were log-transformed to increase normality of the data, though analyses for untransformed and log-transformed durations yielded the same pattern of significance. For the skipping probability, logistic GLMMs were carried out given the binary nature of the dependent variable.

#### Target word analyses

Table 1 shows the means and standard deviations for all the eye movement measures on the target word, and Table 2 shows the corresponding fixed effect estimations for these measures.[Table-anchor tbl1][Table-anchor tbl2]

#### Skipping probability

The difference was reliable between the syntactically infelicitous alternative preview and the identical preview in skipping probability (*b* = −0.12, *SE* = 0.06, *z* = −2.04), but not between the pseudocharacter preview and syntactically infelicitous alternative preview conditions (*b* = −0.09, *SE* = 0.06, *z* = −1.45). However, the difference between the syntactically infelicitous alternative versus identical preview conditions interacted with the target word frequency (*b* = 0.43, *SE* = 0.12, *z* = 3.54). Specifically, for the high-frequency target word, readers skipped the target more often when they had an identical preview compared with the syntactically infelicitous alternative lower frequency preview (*b* = 0.33, *SE* = 0.09, *z* = 3.83), there was no difference between the syntactically infelicitous alternative lower frequency preview and pseudocharacter preview conditions (*b* = 0.03, *SE* = 0.09, *z* = 0.32). For the low-frequency target word, there was no reliable difference between the identical and the syntactically infelicitous alternative higher frequency preview conditions (*b* = −0.09, *SE* = 0.09, *z* = −1.09), however, interestingly, readers were slightly more likely to skip the target when they had a syntactically infelicitous, alternative higher frequency preview compared with a pseudocharacter preview,^1^ although this effect was marginal (*b* = 0.15, *SE* = 0.09, *z* = 1.78, *p* = .07).

Recall that we hypothesized that pretarget launch site (i.e., the number of characters from the location from where a saccade is launched to the start of the target word) might influence parafoveal preview quality, and thus whether an upcoming word may be skipped; skipping effects may be more pronounced for saccades launched closer to the target. To assess whether launch site modulated skipping behavior in this experiment, launch site was centered about its mean (due to it being a continuous variable), and included it in the LMM models as a fixed factor. The fixed effect estimations are shown in Table 3 for all the eye movement measures when launch site distance was included.[Table-anchor tbl3]

There was a reliable three-way interaction between the syntactically infelicitous alternative versus identical preview, target word frequency and launch site distance (*b* = −0.51, *SE* = 0.14, *z* = −3.69). To further investigate this interaction, we used a median split procedure, based on differences in the distance of the saccades to the beginning of the target, classifying contrasting saccades as “near” or “far.” Means and standard deviations for median split launch site data are shown in Table 4.[Table-anchor tbl4]

**Figure 2 fig2:**
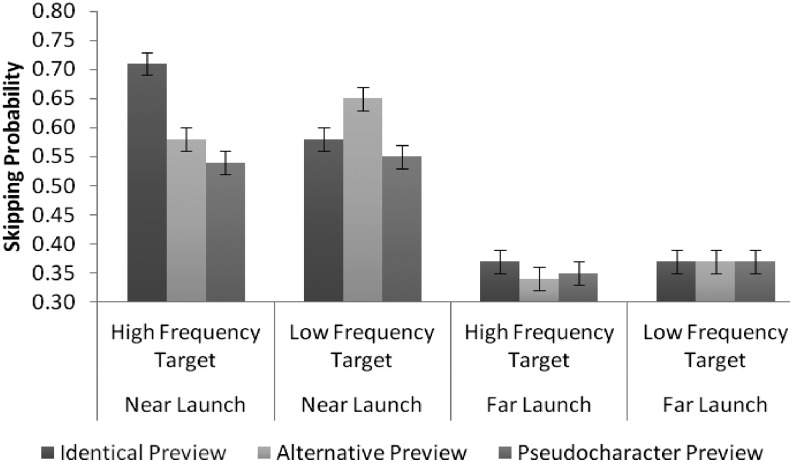
Skipping probability as a function of launch site distance (a median split analyses was applied to the launch site data). Note, Alternative preview refers to syntactically infelicitous alternative preview.

The results showed that when the eyes were launched from a far position, none of the effects was significant, all |z| < 1.21. When the eyes were launched from a near position, however, for the high-frequency target, the skipping rates were higher for identical previews (.71) compared with the syntactically infelicitous alternative lower frequency previews (.58), *b* = 0.69, *SE* = 0.14, *z* = 4.98. There were no differences between the syntactically infelicitous alternative lower frequency preview (.58) and the pseudocharacter preview (.54), *b* = 0.12, *SE* = 0.13, *z* = 0.95. However for the low-frequency target, the skipping rates were higher for the syntactically infelicitous alternative higher frequency preview (.65) compared with the identical previews (.58), *b* = 0.33, *SE* = 0.14, *z* = 2.42, as well as the pseudocharacter preview (.55), *b* = 0.48, *SE* = 0.14, *z* = 3.56 (see Figure 2). These results indicate that readers were more likely to skip a target word with a high-frequency preview—that is, the identical preview for high-frequency targets and the syntactically infelicitous alternative preview for low-frequency targets, than a target word with a low-frequency preview. Thus, readers were still likely to skip a high-frequency preview word even when that word was syntactically infelicitous.[Fig-anchor fig2]

Recall that the decision to skip the target is made prior to the boundary change. This means that from our experimental conditions it is also possible to evaluate how likely it is that a reader will skip a high- or low-frequency (preview) word when it appears in a felicitous compared with an infelicitous syntactic context. For all the target word skipping data, we found no difference between low-frequency, syntactically felicitous previews and low-frequency, syntactically infelicitous previews (*b* = 0.08, *SE* = 0.09, *z* = 0.94), but a marginal difference between high-frequency, syntactically felicitous previews and high-frequency, syntactically infelicitous previews (*b* = 0.16, *SE* = 0.09, *z* = 1.89, *p* = .06), suggesting to a limited extent at least, syntactic felicity may affect readers’ skipping behavior. For the data split between near and far launch sites, the effect was not reliable for low-frequency previews (near: *b* = 0.05, *SE* = 0.13, *z* = 0.41; far: *b* = 0.18, *SE* = 0.13, *z* = 1.40). However, it was reliable for high-frequency previews when the eyes were launched from a near position (*b* = 0.29, *SE* = 0.14, *z* = 2.10), such that readers were more likely to skip the target when they had a high-frequency, syntactically felicitous preview (.71) compared with a high-frequency, syntactically infelicitous preview (.65). However, this effect did not occur when the eyes were launched from a more distant position (*b* = 0.00, *SE* = 0.13, *z* = 0.04). Thus, we obtained a clear demonstration that syntactic context affects word skipping, and that this effect itself is restricted to high-frequency parafoveal words (we will return to this in the General Discussion section), as well as being influenced by the proximity of the eyes to the word to be skipped.

#### Reading times

For reading time measures on the target word, we found a standard preview effect with shorter fixations in the identical condition compared with the syntactically infelicitous alternative preview conditions (all *t* > 6.23). The difference between the syntactically infelicitous alternative preview condition and pseudocharacter preview was not significant (all *t* < 1.15). Furthermore, GD showed a marginal interaction between the syntactically infelicitous alternative versus identical preview conditions and target word frequency (*b* = 0.05, *SE* = 0.03, *t* = 1.65, *p* = .10). Specifically the differences between reading times on the target after the syntactically infelicitous alternative and identical previews were significant for both high- (*b* = 0.12, *SE* = 0.03, *t* = 4.82) and low-frequency targets (*b* = 0.17, *SE* = 0.03, *t* = 6.29), indicating that a syntactically infelicitous visually different preview produced disruption regardless of whether the target word that appeared after the boundary change was high or low frequency.

We also examined whether launch site modulated target word reading times in this experiment. The results showed reliable interactions between launch site and syntactically infelicitous alternative versus identical preview conditions across all reading time measures, and reliable interactions between launch site and pseudocharacter versus alternative preview conditions on GD and go-past times (all *t* > 2.29; see Table 3). Further analyses using a median split between near and far launch sites, showed that the differences between the syntactically infelicitous alternative and identical preview conditions were larger when the eyes were launched from a near position compared to a far position, and these differences in both cases were reliably significant across all the reading time measures (all *t* > 2.71). Furthermore, when the eyes were launched from a near position, GD and go-past times were slightly shorter for syntactically infelicitous alternative preview conditions compared with pseudocharacter previews (GD: *M* = 334 ms, 345 ms for syntactically infelicitous alternative and pseudocharacter previews, respectively, *b* = −0.04, *SE* = 0.02, *t* = −1.65, *p* = .10; go-past time: *M* = 412 ms, 430 ms for syntactically infelicitous alternative and pseudocharacter previews, respectively, *b* = −0.05, *SE* = 0.03, *t* = −1.89, *p* = .06). These results suggest that having a pseudocharacter preview produced more disruption when the target word was processed after the boundary change than having a dissimilar, syntactically infelicitous, but real character preview.

#### Pretarget and posttarget character analyses

Table 5 shows the means of all the eye movement measures on the pretarget and posttarget character, and Table 6 shows the corresponding fixed effect estimations for these measures.[Table-anchor tbl5][Table-anchor tbl6]

For the pretarget character analyses, we found that readers were less likely to skip the pretarget character in the pseudocharacter preview than in the syntactically infelicitous alternative preview condition (*b* = −0.12, *SE* = 0.05, *z* = −2.28), suggesting an orthographic parafoveal-on-foveal effect caused by the presence of a nonsense pseudocharacter in the parafovea. There was no difference between the syntactically infelicitous alternative and identical preview condition in the probability of skipping the pretarget character (*b* = −0.01, *SE* = 0.06, *z* = −0.12). Furthermore, first pass reading measures did not show any reliable effect, but total reading times on the pretarget character were longer in the syntactically infelicitous alternative preview condition than in the identical preview condition (*b* = 0.04, *SE* = 0.02, *t* = 2.39), indicating when readers detected a syntactic infelicity in a visually dissimilar alternative preview, they spent more time rereading the pretarget character.

For our posttarget character analyses, we found that readers were less likely to skip the posttarget character, and spend longer reading it when they had a syntactically infelicitous alternative preview compared with an identical preview (SP: *b* = −0.13, *SE* = 0.05, *z* = −2.40; for reading times, all *t* > 3.84). This is a standard spillover effect. Furthermore, when readers had a syntactically infelicitous alternative preview, FFD, GD, and TFD on the posttarget character were longer than when they had a pseudocharacter preview (FFD: *b* = 0.03, *SE* = 0.01, *t* = 1.92, *p* = .058; GD and TFD: all *t* > 2.02). This suggests that, the syntactically infelicitous visually dissimilar alternative preview caused disruption, and this in turn spilled over to the processing of posttarget characters, both in the early and later stages of post target character processing.

### Discussion

The patterns obtained for all the target word skipping data and those data for saccades launched from a near position are very clear: For the high-frequency target, the probability of skipping the target was higher for the identical preview compared with the syntactically infelicitous alternative lower frequency preview, and there was no difference between the syntactically infelicitous alternative lower frequency preview and the pseudocharacter preview. However, interestingly, the probability of skipping the low-frequency target was higher for the syntactically infelicitous alternative higher frequency preview compared with the identical preview and the pseudocharacter preview. In other words, Chinese readers are more likely to skip a target with a high-frequency preview than a low-frequency preview, even when syntactic context and parafoveal preview information are in conflict. This strongly suggests that skipping a word is very directly influenced by the frequency of the parafoveal word to be skipped, and that such skipping holds for a broad range of high-frequency words and is not restricted to the extremely high-frequency word *de*. Thus, the results of the present study are entirely consistent with, and provide an important extension of the findings of Zang, Zhang et al. (2018) for Chinese reading and the findings of Angele and colleagues (Abbott et al., 2015; Angele et al., 2014; Angele & Rayner, 2013), as well as Choi and Gordon (2013) in English reading.

The follow-up question concerned whether syntactic contextual constraint modulates such skipping of high-frequency words in Chinese reading. The prior research by Zang, Zhang et al. (2018) and Angele and colleagues (Abbott et al., 2015; Angele et al., 2014; Angele & Rayner, 2013) has suggested that syntactic constraints do not modulate skipping decisions. In other words, the decision to skip a Chinese particle *de*, English article *the*, or other higher frequency English word, was mainly based on the available parafoveal information, but not the syntactic structure of the preceding sentence. This conclusion probably holds for extremely high-frequency word skipping. However, both Brothers and Traxler (2016) and Veldre and Andrews (2018a, Experiment 2; see also Snell et al., 2017) have shown that reading times and skipping rates for target words are influenced by the word’s syntactic fit with the preceding sentence. Note that the target words in these alphabetic studies were longer in length, and lower in word frequency compared with those in Angele and colleagues’ studies (Abbott et al., 2015; Angele et al., 2014; Angele & Rayner, 2013), though it was not possible to directly compare the role of syntactic constraint on skipping for high- and low-frequency previews in Angele et al. (2014) as the sentence frames were different for the two comparisons. However, in our study, we could directly compare the probability of skipping a high- or low-frequency preview word when it appeared in a syntactically felicitous versus an infelicitous context. Our results did show that readers were slightly more likely to skip high-frequency words in a syntactically felicitous compared with a syntactically infelicitous context, and this effect was particularly robust when the eyes were launched from a position near to the target word (we will consider this further in the General Discussion section). Based on the current data and those of other existing studies (Brothers & Traxler, 2016; Snell et al., 2017; Veldre & Andrews, 2018a), it appears, to some extent at least, for the high-frequency parafoveal word, its syntactic fit with the context of a sentence plays a role in word skipping during English and Chinese reading.

Next, let us consider the reading time measures on the target words. Standard preview effects were obtained (Li et al., 2015; Zang et al., 2011): Reading times were shorter in the identical preview condition than the syntactically infelicitous dissimilar alternative and pseudocharacter preview conditions, but were similar in the latter two conditions. Furthermore, gaze duration showed a marginal interaction such that the time cost associated with processing a syntactically infelicitous dissimilar preview was slightly greater when the target word was low than high frequency. Presumably, this simply reflects the fact that an inaccurate preview is more costly to the identification process (i.e., it sets the process back more) when a word is harder to identify.

At the pretarget character region, we obtained an orthographic parafoveal-on-foveal effect on skipping of the target with a lower skipping rate for the pseudocharater preview compared with a syntactically infelicitous alternative real character preview. Clearly, there was sensitivity to a visually unfamiliar pseudocharacter prior to a saccade to it or beyond it. In contrast, there was no evidence in fixation durations on the pretarget word to suggest a parafoveal-on-foveal syntactic infelicity effect. Times were similar for syntactically infelicitous and syntactically felicitous previews. Similar results were reported in Brothers and Traxler (2016), Snell et al. (2017), and Veldre and Andrews (2018a). Interestingly, the posttarget character analyses did show strong effects of disruption from the syntactically infelicitous alternative preview, relative to the felicitous identity condition. This effect was robust both on the early and late processing measures and suggests that the infelicity not only affected skipping behavior in relation to the target, but also fixation durations on the target as well as regions downstream from the target. Thus, infelicity effects not only have an early influence on eye movements, but that influence is sustained through processing until fairly late stages after the eyes leave the word. To be clear, it appears that syntactic infelicity effects are immediate, robust, and have an extended time course during reading.

It should be noted that in the boundary paradigm (Rayner, 1975), when readers’ eyes cross an invisible boundary, the previews change to the target word. Because the change occurs during a saccade, readers are generally unaware of it. However, as is often reported in recent boundary studies, there are individual differences in display change awareness with some participants being more likely to be aware than others. Previous research has shown that the size of the preview effect is generally larger for the more than for the less aware participants (e.g., Angele, Slattery, & Rayner, 2016; Slattery, Angele, & Rayner, 2011; Veldre & Andrews, 2018c; White, Rayner, & Liversedge, 2005b), however, it is also the case that the more aware participants might produce a qualitatively different data pattern from those who are less aware of the display changes (see Angele et al., 2016; Veldre & Andrews, 2018c; White et al., 2005b for discussion of change awareness, foveal load and parafoveal processing that we will not discuss here). Veldre and Andrews (2018c) argued that task differences might account for differences in awareness. For instance, in one task participants were required to indicate whether they had noticed any changes in the sentences they were reading after completion of the experiment, and if so, to estimate the overall number of changes that they had noticed (postexperiment estimation, e.g., White et al., 2005b). In an alternative task participants were asked after every trial if they detected a display change (every-trial detection, e.g., Angele et al., 2016; Slattery et al., 2011). In the every-trial detection task, participants remained alert to the possibility of changes from the beginning to the end of the experiment, and this may have affected the nature of their reading strategy. In Experiment 2, we were primarily interested in investigating whether display change awareness influenced the patterns of results that we obtained in Experiment 1, and whether these effects were modulated by different change detection tasks.

## Experiment 2

### Method

#### Participants

Seventy-nine undergraduate students (mean age = 20 years, *SD* = 2.3) from Tianjin Normal University, participated in the experiment. Of these, 36 completed the postexperiment estimation task, and the rest of them completed the every-trial detection task. None of them had participated in Experiment 1.

#### Apparatus and materials

Identical to Experiment 1.

#### Procedure

The sentences were presented in the same way as in Experiment 1. The critical difference from Experiment 1 was that, in the postexperiment estimation task, participants were asked at the end of the experiment, if they had noticed any changes regarding the display of the sentences they were reading, and if so, to estimate how many display changes they had noticed. In contrast, in the every-trial detection task, participants were asked after every trial, to indicate whether they noticed any changes on the screen and press a yes/no response key on the button box. Occasionally they were presented with comprehension questions after some of the sentences, and these questions were presented immediately after the display change detection screen.

### Results

Overall, the mean comprehension accuracy of participants was 93%, indicating they understood the sentences well. There were differences in comprehension accuracy between participants who were in the postexperiment estimation task (*M* = 96%, range = 88%–100%) and those in the every-trial detection task (*M* = 91%, range = 80%–100%), *t* = 4.85, *p* < .01. This suggests that comprehension might be slightly more difficult for participants in the latter compared to the former task, replicating the observation reported by Vasilev, Slattery, Kirkby, and Angele (2018). We applied the same data exclusion criteria as in Experiment 1. Fixations shorter than 80 ms or longer than 1,200 ms were excluded from the analyses. Trials were removed prior to analysis if (a) a track loss occurred or there were fewer than three fixations in total (0.22%); (b) eye movement measures were above or below three standard deviations from each participant’s mean (1.5%); (c) a blink occurred during display changes or during a fixation on the target word, as well as trials in which the display changes occurred in an untimely or delayed manner (6.2%).

#### Change detection task

We first conducted LMM analyses including fixed effects, change detection task, target word frequency, and preview condition, as well as their interactions to investigate whether change detection task influence target word processing. Table 7 shows the means and standard deviations for all the eye movement measures on the target word, and Table 8 shows the corresponding fixed effect estimations for these measures.[Table-anchor tbl7][Table-anchor tbl8]

#### Skipping probability

The effect of change detection task was not reliable (*b* = 0.01, *SE* = 0.17, *z* = 0.03), but it interacted with frequency in skipping probability (*b* = 0.30, *SE* = 0.09, *z* = 3.27). However follow-up tests indicated that the difference between the two tasks was not statistically significant for either high- or low-frequency words (all *z* < 1). Change detection task did not interact with any other variables; therefore we obtained no evidence to indicate that it influences effects on skipping behavior.^2^

Importantly, similar to Experiment 1, skipping rates were higher for the identical preview than the syntactically infelicitous alternative preview (*b* = 0.15, *SE* = 0.06, *z* = 2.68), and higher for the syntactically infelicitous alternative preview than the pseudocharacter preview (*b* = 0.23, *SE* = 0.06, *z* = 3.95). Furthermore, there was a reliable interaction between the syntactically infelicitous alternative versus identical preview and word frequency (*b* = 0.53, *SE* = 0.11, *z* = 4.68). Specifically, readers skipped the high-frequency target more often when they had an identical preview compared with the syntactically infelicitous alternative lower frequency preview (*b* = 0.41, *SE* = 0.08, *z* = 5.18). For the low-frequency target, there was a numerical difference between the identical and syntactically infelicitous alternative higher frequency previews, but this was not statistically reliable (*b* = −0.10, *SE* = 0.08, *z* = −1.28). Using a median split method to classify contrasting saccades as “near” or “far” as per Experiment 1, the results showed when the eyes were launched from a far position, none of the effects was significant, all |z| < 1; when the eyes were launched from a near position, for the high-frequency target, the skipping rates were higher for the identical previews (.78) compared with the syntactically infelicitous alternative lower frequency previews (.62), *b* = 0.95, *SE* = 0.13, *z* = 7.33. However for the low-frequency target,^3^ the skipping rates were numerically higher for the syntactically infelicitous alternative higher frequency preview (.70) compared with the identical preview (.68), *b* = 0.21, *SE* = 0.13, *z* = 1.66, *p <* .10. This effect was marginal. These results suggest that a word with a high-frequency preview is slightly more likely to be skipped than that with a low-frequency preview, and the pattern of effects overall is in line with the findings from Experiment 1.

To further assess the role of syntactic felicity in skipping we also compared the high-frequency, syntactically felicitous preview with the high-frequency, syntactically infelicitous preview, and the low-frequency, syntactically felicitous preview and the low-frequency, syntactically infelicitous preview, respectively. The results showed significant effects for the former (*b* = 0.23, *SE* = 0.08, *z* = 2.82) but not for the latter comparison (*b* = 0.08, *SE* = 0.08, *z* = 1.06). For the data split between near and far launch sites, when the eyes were launched from a near position, the former comparison was very reliable with higher skipping rates for the high-frequency, syntactically felicitous preview (.78) than for the high-frequency, syntactically infelicitous preview (.70), *b* = 0.48, *SE* = 0.13, *z* = 3.63, and the latter comparison was marginal with higher skipping rates for the low-frequency, syntactically felicitous preview (.68) than for the low-frequency, syntactically infelicitous preview (.62), *b* = 0.23, *SE* = 0.12, *z* = 1.89, *p* = .06. No robust effects occurred when the eyes were launched from a far position, all *z* < 1. Again, these results entirely replicate the findings from Experiment 1 and demonstrate a limited role of syntactic context in word skipping.

#### Reading times

Compared with the postexperiment estimation task, readers spent longer reading in the every-trial detection task as indicated by marginal effects on FFD, GD, and go-past times (all *t* > 1.78, *p* = .07) and a significant effect on TFD (*b* = 0.13, *SE* = 0.05, *t* = 2.75). Interestingly, the change detection task did not interact with any other conditions on reading times. Consistent with the comprehension accuracy data, these results demonstrate that the every-trial detection task might be more difficult for participants and thus cause them to spend a longer time reading. However, critically, there was no evidence to show that the type of change detection participants engaged in modulated target reading time effects.

As in Experiment 1, reading times on the target were longer after a syntactically infelicitous alternative preview compared to an identical preview (all *t* > 4.19), and slightly longer in the pseudocharacter preview compared with the syntactically infelicitous alternative preview condition on GD (*b* = 0.03, *SE* = 0.02, *t* = 1.78, *p* = .08) and TFD (*b* = 0.03, *SE* = 0.02, *t* = 1.90, *p* = .06), which replicated the standard preview effect. We obtained a reliable frequency effect on go-past times (*b* = 0.04, *SE* = 0.01, *t* = 2.69) and TFD (*b* = 0.03, *SE* = 0.02, *t* = 2.17) with longer times for the low- compared with the high-frequency target. Moreover, frequency interacted with the syntactically infelicitous alternative versus identical preview on FFD, GD, and go-past times (all *t* > 2.85), as well as with the pseudocharacter versus syntactically infelicitous alternative preview on go-past times (*b* = 0.08, *SE* = 0.04, *t* = 2.15). Further tests showed that the frequency effect was reliable in the identical (all *t* > 3.23) rather than the syntactically infelicitous alternative preview conditions (all *t* < 1.40). Finally, for high-frequency targets, go-past times were similar when readers had a syntactically infelicitous alternative lower frequency preview compared with a pseudocharacter preview (*b* = 0.01, *SE* = 0.02, *t* = 0.47). Whereas for low-frequency targets, go-past times were longer when readers had a pseudocharacter preview compared with a higher frequency syntactically infelicitous preview (*b* = 0.07, *SE* = 0.03, *t* = 2.59), results that are, again, comparable with the standard preview effect (Rayner, 1998, 2009; Zang et al., 2011). Overall, these results are very consistent with those we reported in Experiment 1, revealing that these effects were not influenced by the display change detection task.

#### Display change awareness

Display change awareness was defined as the proportion of trials on which a display change was detected and reported. Using a median split method, in the postexperiment estimation task, 18 participants reported noticing changes in more than 10 sentences (more aware participants), and 18 reported noticing changes in less than 10 sentences (less aware participants). For the every-trial detection task, 22 participants reported noticing changes in more than 14 sentences (more aware participants) and 21 participants reported noticing changes in less than 14 sentences (less aware participants).^4^ Because of the lack of evidence indicating that the nature of the change detection task modulated the patterns of results that occurred in Experiment 1, we first collapsed data over change detection task, and then included display change awareness (more or less aware) as a variable in the LMM analyses, to formally investigate the potential influence of display change awareness. The means and standard deviations for all the eye movement measures on the target word are presented in Table 9, and the results from the LMMs are presented in Table 10.[Table-anchor tbl9][Table-anchor tbl10]

Compared with Tables 2 and 8, Table 10 shows very similar patterns. For this reason, and for brevity’s sake, we do not provide a comprehensive description of all the patterns of results. Instead, here we only focus on the effect of display change awareness and its interaction with other variables. Clearly, in contrast to the less aware participants, more aware participants showed reduced skipping rates (*b* = −0.66, *SE* = 0.15, *z* = −4.34) and spent a longer time on the target word (all *t* > 2.61), suggesting that they were reading more carefully and perhaps more cautiously. Importantly, display change awareness did not interact with any other variables on skipping.^5^ However it did interact with syntactically infelicitous alternative versus identical preview on GD (*b* = 0.10, *SE* = 0.04, *t* = 2.63), go-past times (*b* = 0.09, *SE* = 0.05, *t* = 1.73, *p* = .09), and TFD (*b* = 0.11, *SE* = 0.04, *t* = 2.44), and with pseudocharacter versus syntactically infelicitous alternative preview on go-past times (*b* = 0.10, *SE* = 0.04, *t* = 2.31). Further contrast analyses showed that all the differences were reliable between the syntactically infelicitous alternative and identical previews for both less and more aware participants (GD–less aware: *b* = 0.07, *SE* = 0.02, *t* = 3.30; GD–more aware: *b* = 0.18, *SE* = 0.02, *t* = 8.78; go-past–less aware: *b* = 0.13, *SE* = 0.03, *t* = 4.36; go-past–more aware: *b* = 0.22, *SE* = 0.02, *t* = 9.20; TFD–less aware: *b* = 0.05, *SE* = 0.02, *t* = 2.25; TFD–more aware: *b* = 0.16, *SE* = 0.02, *t* = 7.81); the preview effects were larger for participants who were more likely to be aware of the display change than for those who were less likely to be aware. Similar patterns occurred for the pseudocharacter versus syntactically infelicitous alternative previews for both the less aware and the more aware participants. All the results replicate the previous literature (Angele et al., 2016; Slattery et al., 2011; Veldre & Andrews, 2018c; White et al., 2005b), which showed increased display change awareness was associated with increased preview effects, but it did not change the pattern of effects over different preview conditions.

Finally, neither change detection task, nor display change awareness, altered the basic patterns of eye movements on pretarget and posttarget characters, and these data are, therefore, not reported.

### Discussion

Experiment 2 was conducted primarily to assess whether or not the nature of the display change detection task, or display change awareness more generally, might lead to changes in the patterns of skipping and reading time reported in Experiment 1. The results are robust and straightforward: Readers spent longer reading the text when they were required to detect changes after every trial than when they were required to estimate change awareness after completion of the experiment. Furthermore, readers who were more likely to be aware of changes skipped the target less and spent longer reading the target than those who were less likely to be aware of changes. However, there was no reliable evidence to demonstrate that the high-frequency preview and syntactic felicity effects that we observed in Experiment 1 depended on either display change awareness or the specific change detection task. In other words, the findings from Experiment 2 entirely replicate the basic pattern of skipping and reading time results from Experiment 1 with some effects (e.g., frequency), if anything, being even more pronounced. To reiterate, neither the display change detection task, nor display change awareness influenced the nature of these effects.

## General Discussion

We have reported two experiments to investigate whether the *de*-skipping effect reported by Zang, Zhang et al. (2018) was specific to the high-frequency particle *de*, or it was associated with a broader range of other high-frequency words, and to further determine the relative importance of parafoveal processing and syntactic context in word skipping during Chinese reading. In the Zang, Zhang et al. (2018) study, the critical finding was that when the upcoming word in the parafovea was the high-frequency particle *de*, even when this word was incompatible with the sentential syntactic context, Chinese readers were still more likely to skip it, compared with the correct preview of the target which was compatible with the syntactic sentence context.

In Experiment 1, we manipulated target word frequency (high or low) and preview using the gaze-contingent boundary paradigm. The preview of the target word was either identical to the target, a nonsense pseudocharacter, or a lower or higher frequency alternative that was syntactically infelicitous with respect to the preceding sentence context. Experiment 2 was very similar to Experiment 1, in which the nature of the contingent change detection task, and readers’ display change awareness were considered. Both experiments clearly demonstrate that the *de*-skipping effect generalizes to other high-frequency words during Chinese reading. The patterns obtained for all the target word skipping data are very straightforward: When readers have a high-frequency preview, even if it is in conflict with the syntactic context, they are still more likely to skip it compared with when they have a low-frequency preview. These effects occurred in Experiment 1 and were entirely consistent in Experiment 2. The results indicate that word skipping is strongly influenced by the familiarity of a parafoveal word, thus extending effects demonstrated for the extremely high-frequency Chinese word *de*, or the English word *the*, to other high-frequency words in Chinese.

To further examine the role of syntactic contextual constraint in word skipping during Chinese reading, we directly compared the probability of skipping a high- or low-frequency preview when it appeared in a syntactically felicitous versus an infelicitous context. Both experiments demonstrated a reliable syntactic felicity effect for the high-frequency preview, and Experiment 2 also showed a marginally significant syntactic felicity effect for the low-frequency preview when the eyes were launched from a position near to that word. To some extent at least, a word’s syntactic fit with the sentential context plays a role in word skipping during Chinese reading, and this is especially the case when those parafoveal words are high frequency. We mentioned earlier that some other researchers have shown that word skipping is influenced by a word’s syntactic fit with the previous context (Brothers & Traxler, 2016; Snell et al., 2017; Veldre & Andrews, 2018a, Experiment 2 for plausible context), while some other researchers have not (Angele et al., 2014; Angele & Rayner, 2013), and differences in parafoveal word length (and therefore frequency) may contribute to the diverging findings. Because researchers did not directly compare the role of syntactic context in skipping for high- and low-frequency previews, it was assumed that lexical familiarity appeared to take precedence for skipping of short function words (like *the* in English), whereas in other circumstances syntactic context played an overriding role. Thus, Brothers and Traxler (2016) argued that syntactic constraint effects are anticipatory in nature such that readers use them to preactive word-class information for parafoveal words, resulting in higher skipping rates for a syntactically congruent preview relative to a syntactically incongruent preview.

According to Brothers and Traxler (2016), syntactic information can be activated rapidly and thus can influence the earliest stage of word identification in reading of alphabetic languages. The current study allows us to form conclusions beyond this in that the syntactic felicity effect we observed in skipping was more pronounced for high-frequency previews relative to low-frequency previews. This together with our finding that skipping effects occurred for high-frequency previews even when they were syntactically infelicitous, leads us to argue that when the upcoming word is extremely high frequency (e.g., *de* in Chinese), then its identification in the parafovea is automatic regardless of the preceding syntactic context. However, when the upcoming word is lower frequency, both its lexical familiarity based on visual information extracted from the parafovea, and sentential syntactic constraints (at least to a limited degree) jointly influence the decision to skip during reading. In other words, at least for Chinese reading, it appears that the lexical frequency of an upcoming word mediates the influence of syntactic information on the formulation of a decision to skip that word. We tentatively suggest that these effects might be particularly prominent in Chinese because the language is orthographically dense (see Liversedge et al., 2016), lacks syntactic markers and is unspaced (with consequent increased lateral masking). All these factors very likely contribute to the temporal extension of the time course of lexical processing in natural reading (again, see Liversedge et al., 2016 for related arguments).

Both the E-Z Reader model (Reichle, 2011; Reichle et al., 2003; Reichle et al., 2009) and the SWIFT model (Engbert & Kliegl, 2011; Engbert et al., 2005; Schad & Engbert, 2012) are able to account for our findings with regard to skipping and the lexical frequency of upcoming words, as both assume that lexical familiarity of parafoveal words indexed by frequency, as well as the predictability of a word based on sentence context, strongly influence saccade targeting during reading. Notably, however, E-Z Reader 10 (Reichle, 2011; Reichle et al., 2009) also includes a postlexical integration stage of processing (*I*) to account for how higher-level, postlexical, linguistic factors influence eye movements. It posits that the stage *I* begins after the completion of the identification of a word. In other words, lexical processing strictly precedes the process by which a word is integrated into the sentential syntactic and semantic context. Staub (2011) manipulated word frequency and the syntactic fit of a word with respect to context and conducted a series of simulations to examine the measurable effects on eye movements. Staub (2011) found that integration difficulty associated with a syntactic fit manipulation influenced early fixation times such as first fixation duration on target words, but did not influence skipping rates, except for very short words which could be rapidly identified in the parafovea. Similar findings were obtained by Abbott and Staub (2015) with frequency, but not plausibility influencing word skipping. However, Veldre and Andrews (2018a) found plausibility preview effects on skipping rates (see also Veldre & Andrews, 2017, 2018b). As we better understand the influence of syntactic context, as well as other sources of processing constraint, in relation to eye movements in reading, the models will require further specification.

To summarize, the increased skipping effect is not specific to the particle *de* in Chinese reading, but is also associated with other high-frequency words with different syntactic categories. When parafoveal preview of a word indicates that a word is familiar and high frequency, even if it may not fit with the syntactic context of the sentence within which it appears, readers are still likely to skip it. Thus, at least for high-frequency Chinese words, lexical familiarity and syntactic felicity of a parafoveal preview have a substantial influence on whether or not an upcoming word is skipped. However, it is also the case that, to a limited degree, the syntactic felicity of less familiar words can also influence fixation durations on them and fixations downstream from them (Brothers & Traxler, 2016; Snell et al., 2017; Veldre & Andrews, 2018a).

## Figures and Tables

**Table 1 tbl1:** Eye Movement Measures for the Target Word

Measure	High frequency			Low frequency		
	Identical	Syntactically infelicitous alternative	Pseudocharacter	Identical	Syntactically infelicitous alternative	Pseudocharacter
SP	.53 (.50)	.46 (.50)	.45 (.50)	.48 (.50)	.50 (.50)	.47 (.50)
FFD	253 (93)	288 (119)	291 (117)	252 (87)	291 (117)	292 (122)
GD	259 (101)	305 (138)	313 (135)	257 (94)	316 (146)	323 (145)
Go-past	304 (180)	386 (245)	379 (221)	298 (180)	378 (209)	403 (247)
TFD	298 (155)	352 (198)	352 (188)	307 (166)	357 (194)	363 (207)
*Note*. Standard deviations are provided in parentheses. SP = skipping probability; FFD = first fixation duration; GD = gaze duration; Go-past = go-past time; TFD = total fixation duration.						

**Table 2 tbl2:** LMM Analyses on the Target Word

Factor	SP			FFD			GD			Go-past			TFD		
	*b*	*SE*	*z*	*b*	*SE*	*t*	*b*	*SE*	*t*	*b*	*SE*	*t*	*b*	*SE*	*t*
(Intercept)	−.11	.08	−1.34	5.53	.02	**269**	5.58	.02	**236**	5.72	.03	**208**	5.67	.03	**214**
Preview															
Syntactically infelicitous alternative versus identical	−.12	.06	**−2.04**	.11	.02	**6.36**	.14	.02	**7.03**	.20	.03	**7.59**	.13	.02	**6.24**
Pseudocharacter versus syntactically infelicitous alternative	−.09	.06	−1.45	−.00	.01	−.16	.02	.02	1.14	.01	.02	.49	.01	.02	.62
Frequency: High versus low	−.01	.05	−.16	.00	.01	.22	.01	.01	1.02	.01	.02	.43	.01	.01	1.03
Interactions															
Syntactically Infelicitous Alternative Versus Identical × Frequency	.43	.12	**3.54**	.03	.03	1.01	.05	.03	1.65	.03	.04	.87	−.01	.03	−.28
Pseudocharacter Versus Syntactically Infelicitous Alternative × Frequency	−.13	.12	−1.07	−.03	.03	−1.01	−.03	.03	−.97	.03	.04	.79	−.01	.03	−.25
*Note*. Significant terms are marked in bold, and marginally significant items are underlined. *b* = regression coefficient.															

**Table 3 tbl3:** LMM Analyses on the Target Word When Launch Site Was Included as a Variable

Factor	SP			FFD			GD			Go-past			TFD		
	*b*	*SE*	*z*	*b*	*SE*	*t*	*b*	*SE*	*t*	*b*	*SE*	*t*	*b*	*SE*	*t*
(Intercept)	−.15	.13	−1.15	5.53	.02	**276**	5.58	.02	**245**	5.71	.03	**208**	5.67	.03	**212**
Preview															
Syntactically infelicitous alternative versus identical	−.14	.07	**−2.18**	.12	.02	**6.80**	.15	.02	**7.59**	.21	.03	**8.17**	.13	.02	**6.75**
Pseudocharacter versus syntactically infelicitous alternative	−.13	.07	**−2.02**	−.00	.01	−.33	.01	.02	.89	.01	.02	.35	.01	.02	.58
Frequency: High versus low	.01	.05	.28	.00	.01	.31	.01	.01	1.13	.01	.02	.59	.01	.01	1.00
Launch site	−.84	.03	**−26.2**	−.01	.01	**−2.67**	−.02	.01	**−4.51**	.00	.01	.67	.01	.01	1.73
Interactions															
Syntactically Infelicitous Alternative Versus Identical × Frequency	.42	.13	**3.19**	.03	.03	.96	.05	.03	1.63	.03	.04	.73	−.01	.03	−.32
Pseudocharacter Versus Syntactically Infelicitous Alternative × Frequency	−.12	.13	−.93	−.03	.03	−1.09	−.03	.03	−1.06	.03	.03	.80	−.01	.03	−.32
Syntactically Infelicitous Alternative Versus Identical × Launch Site	.09	.07	1.26	−.04	.01	**−3.01**	−.03	.01	**−2.78**	−.05	.02	**−3.20**	−.03	.01	**−2.35**
Pseudocharacter Versus Syntactically Infelicitous Alternative × Launch Site	.11	.07	1.62	−.00	.01	−.05	−.03	.01	**−2.30**	−.04	.01	**−2.39**	−.02	.01	−1.23
Frequency × Launch Site	.12	.05	**2.24**	−.01	.01	−.93	−.01	.01	−1.40	−.01	.01	−1.18	−.01	.01	−1.02
Syntactically Infelicitous Alternative Versus Identical × Frequency × Launch Site	−.51	.14	**−3.69**	.02	.02	1.02	.01	.02	.59	.01	.03	.32	−.01	.03	−.19
Pseudocharacter Versus Syntactically Infelicitous Alternative × Frequency × Launch Site	.22	.13	1.63	−.02	.02	−.97	−.02	.02	−.93	.01	.03	.18	−.03	.03	−1.21
*Note*. Significant terms are marked in bold, and marginally significant items are underlined. *b* = regression coefficient.															

**Table 4 tbl4:** Eye Movement Measures on the Target Word When a Median Split Procedure Was Applied to the Launch Site Data

	Launch site = Near						Launch site = Far					
	High frequency			Low frequency			High frequency			Low frequency		
Measure	Identical	Syntactically infelicitous alternative	Pseudocharacter	Identical	Syntactically infelicitous alternative	Pseudocharacter	Identical	Syntactically infelicitous alternative	Pseudocharacter	Identical	Syntactically infelicitous alternative	Pseudocharacter
SP	.71 (.46)	.58 (.49)	.54 (.50)	.58 (.49)	.65 (.48)	.55 (.50)	.37 (.48)	.34 (.47)	.35 (.48)	.37 (.48)	.37 (.48)	.37 (.48)
FFD	243 (91)	306 (130)	302 (130)	249 (87)	305 (130)	310 (137)	260 (94)	269 (103)	278 (98)	254 (88)	281 (105)	272 (98)
GD	252 (105)	326 (151)	335 (153)	258 (96)	341 (169)	355 (163)	265 (98)	284 (121)	287 (102)	257 (91)	297 (121)	285 (110)
Go-Past	287 (177)	405 (252)	418 (245)	292 (170)	416 (240)	444 (257)	317 (181)	366 (236)	332 (177)	304 (189)	349 (178)	356 (225)
TFD	290 (164)	368 (217)	367 (214)	311 (177)	377 (216)	389 (225)	305 (148)	333 (170)	334 (147)	304 (157)	339 (170)	332 (180)

**Table 5 tbl5:** Eye Movement Measures for the Pretarget and Posttarget Character

Measure	High frequency			Low frequency		
	Identical	Syntactically infelicitous alternative	Pseudocharacter	Identical	Syntactically infelicitous alternative	Pseudocharacter
Pretarget character						
SP	.57 (.50)	.58 (.49)	.55 (.50)	.59 (.49)	.58 (.49)	.55 (.50)
FFD	223 (71)	228 (76)	227 (72)	225 (67)	225 (79)	227 (70)
GD	226 (76)	235 (93)	235 (91)	231 (78)	232 (93)	233 (87)
Go-past	271 (164)	267 (156)	267 (158)	260 (140)	271 (159)	265 (151)
TFD	273 (128)	301 (172)	295 (170)	275 (143)	299 (174)	304 (176)
Posttarget character						
SP	.54 (.50)	.53 (.50)	.52 (.50)	.54 (.50)	.50 (.50)	.53 (.50)
FFD	239 (83)	252 (93)	245 (88)	238 (82)	253 (92)	248 (92)
GD	246 (93)	261 (102)	252 (93)	240 (84)	264 (106)	259 (105)
Go-past	297 (185)	362 (214)	346 (203)	303 (184)	387 (248)	368 (234)
TFD	289 (155)	305 (160)	300 (153)	289 (145)	315 (158)	300 (160)

**Table 6 tbl6:** LMM Analyses for the Pretarget and Posttarget Character

	SP			FFD			GD			Go-past			TFD		
Factor	*b*	*SE*	*z*	*b*	*SE*	*t*	*b*	*SE*	*t*	*b*	*SE*	*t*	*b*	*SE*	*t*
Pretarget character															
(Intercept)	.30	.06	**5.11**	5.36	.02	**328**	5.38	.02	**300**	5.47	.02	**230**	5.53	.02	**249**
Preview															
Syntactically infelicitous alternative versus identical	−.01	.06	−.12	.00	.01	.22	.00	.01	.25	−.01	.02	−.41	.04	.02	**2.39**
Pseudocharacter versus syntactically infelicitous alternative	−.12	.05	**−2.28**	.01	.01	.87	.01	.01	.80	.01	.02	.60	.01	.02	.33
Frequency: High versus low	.04	.04	.85	.00	.01	.06	.00	.01	.01	−.00	.01	−.17	.00	.01	.05
Interactions															
Syntactically Infelicitous Alternative Versus Identical × Frequency	−.10	.11	−.87	−.03	.02	−1.26	−.04	.02	−1.59	.01	.03	.40	−.01	.03	−.46
Pseudocharacter Versus Syntactically Infelicitous Alternative × Frequency	−.02	.11	−.18	.02	.02	.98	.01	.02	.60	.02	.03	.62	.03	.03	1.11
Posttarget character															
(Intercept)	.11	.05	**2.06**	5.43	.02	**337**	5.46	.02	**332**	5.68	.02	**244**	5.57	.02	**261**
Preview															
Syntactically infelicitous alternative versus identical	−.13	.05	**−2.40**	.06	.01	**4.37**	.07	.01	**4.97**	.18	.02	**8.32**	.07	.02	**3.85**
Pseudocharacter versus syntactically infelicitous alternative	.04	.05	.79	−.03	.01	−1.92	−.03	.01	**−2.03**	−.02	.02	−1.14	−.04	.02	**−2.30**
Frequency: High versus low	−.02	.04	−.56	.01	.01	.60	.01	.01	.53	.04	.02	**2.07**	.01	.01	.39
Interactions															
Syntactically Infelicitous Alternative Versus Identical × Frequency	−.09	.11	−.85	.00	.02	.03	.01	.03	.54	.03	.04	.79	.02	.03	.66
Pseudocharacter Versus Syntactically Infelicitous Alternative × Frequency	.13	.11	1.24	.00	.02	.13	.01	.03	.47	−.01	.04	−.24	−.04	.03	−1.18
*Note*. Significant terms are marked in bold, and marginally significant items are underlined. *b* = regression coefficient.															

**Table 7 tbl7:** Eye Movement Measures on the Target Word When Change Detection Task Was Included as a Variable

	Postexperiment estimation task						Every-trial detection task					
	High frequency			Low frequency			High frequency			Low frequency		
Measure	Identical	Syntactically infelicitous alternative	Pseudocharacter	Identical	Syntactically infelicitous alternative	Pseudocharacter	Identical	Syntactically infelicitous alternative	Pseudocharacter	Identical	Syntactically infelicitous alternative	Pseudocharacter
SP	.61 (.46)	.51 (.48)	.46 (.47)	.47 (.48)	.53 (.47)	.46 (.45)	.56 (.48)	.48 (.44)	.45 (.45)	.54 (.47)	.54 (.48)	.49 (.46)
FFD	242 (62)	265 (84)	271 (99)	254 (75)	266 (93)	270 (95)	244 (74)	291 (96)	290 (97)	261 (74)	284 (97)	296 (107)
GD	251 (71)	284 (101)	291 (107)	266 (87)	287 (107)	296 (110)	250 (82)	313 (110)	317 (111)	276 (90)	314 (119)	324 (126)
Go-past	298 (137)	377 (221)	367 (182)	328 (143)	358 (165)	380 (177)	316 (156)	407 (190)	401 (178)	356 (175)	407 (202)	409 (188)
TFD	293 (133)	317 (140)	321 (147)	303 (130)	333 (155)	354 (165)	325 (159)	386 (183)	394 (181)	340 (150)	391 (187)	395 (190)

**Table 8 tbl8:** LMM Analyses on the Target Word When Change Detection Task Was Included as a Variable

	SP			FFD			GD			Go-past			TFD		
Factor	*b*	*SE*	*z*	*b*	*SE*	*t*	*b*	*SE*	*t*	*b*	*SE*	*t*	*b*	*SE*	*t*
(Intercept)	.04	.09	.46	5.53	.02	**359**	5.58	.02	**317**	5.76	.03	**228**	5.71	.02	**234**
Preview															
Syntactically infelicitous alternative versus identical	−.15	.06	**−2.68**	.09	.02	**4.86**	.12	.02	**5.76**	.16	.03	**6.18**	.10	.02	**4.20**
Pseudocharacter versus syntactically infelicitous alternative	−.23	.06	**−3.95**	.01	.01	.90	.03	.02	1.78	.02	.02	.90	.03	.02	1.90
Frequency: High versus low	−.04	.05	−.78	.01	.01	1.08	.02	.01	1.61	.04	.01	**2.69**	.03	.02	**2.17**
Task	.01	.17	.03	.05	.03	1.83	.06	.03	1.79	.09	.05	1.81	.13	.05	**2.75**
Interactions															
Syntactically Infelicitous Alternative Versus Identical × Frequency	.53	.11	**4.68**	−.09	.03	**−3.12**	−.10	.03	**−2.86**	−.14	.04	**−3.88**	−.03	.03	−.88
Pseudocharacter Versus Syntactically Infelicitous Alternative × Frequency	−.10	.11	−.86	.04	.03	1.26	.04	.03	1.14	.08	.04	**2.15**	.01	.03	.36
Syntactically Infelicitous Alternative Versus Identical × Task	−.05	.11	−.44	.04	.03	1.16	.05	.04	1.33	.03	.05	.61	.07	.05	1.50
Pseudocharacter Versus Syntactically Infelicitous Alternative × Task	.03	.11	.31	.03	.03	1.17	.02	.03	.51	.03	.04	.59	−.01	.03	−.29
Frequency × Task	.30	.09	**3.27**	−.00	.02	−.01	−.00	.02	−.07	−.02	.03	−.70	−.03	.03	−1.16
Syntactically Infelicitous Alternative Versus Identical × Frequency × Task	−.33	.23	−1.47	−.03	.06	−.47	−.03	.07	−.47	.01	.07	.11	−.04	.07	−.62
Pseudocharacter Versus Syntactically Infelicitous Alternative × Frequency × Task	−.04	.23	−.19	−.00	.06	−.01	−.01	.06	−.15	−.03	.07	−.35	−.06	.06	−.90
*Note*. Significant terms are marked in bold, and marginally significant items are underlined. *b* = regression coefficient.															

**Table 9 tbl9:** Eye Movement Measures on the Target Word When Display Change Awareness Was Included as a Variable

	Less aware participants						More aware participants					
	High frequency			Low frequency			High frequency			Low frequency		
Measure	Identical	Syntactically infelicitous alternative	Pseudocharacter	Identical	Syntactically infelicitous alternative	Pseudocharacter	Identical	Syntactically infelicitous alternative	Pseudocharacter	Identical	Syntactically infelicitous alternative	Pseudocharacter
SP	.64 (.46)	.57 (.47)	.55 (.47)	.57 (.46)	.62 (.47)	.57 (.46)	.54 (.49)	.42 (.45)	.37 (.45)	.46 (.48)	.47 (.47)	.39 (.46)
FFD	236 (59)	262 (82)	258 (83)	250 (72)	266 (85)	271 (92)	249 (78)	293 (98)	301 (111)	265 (77)	285 (104)	295 (109)
GD	247 (69)	273 (87)	271 (91)	260 (80)	278 (91)	285 (103)	254 (84)	324 (122)	335 (125)	281 (96)	323 (133)	334 (132)
Go-past	302 (126)	370 (187)	341 (162)	333 (156)	367 (185)	333 (148)	314 (166)	415 (219)	425 (196)	352 (164)	401 (186)	450 (211)
TFD	304 (142)	313 (138)	313 (140)	300 (124)	325 (156)	343 (169)	316 (151)	391 (186)	403 (188)	344 (155)	399 (187)	406 (188)

**Table 10 tbl10:** LMM Analyses on the Target Word When Display Change Awareness Was Included as a Variable

	SP			FFD			GD			Go-past			TFD		
Factor	*b*	*SE*	*z*	*b*	*SE*	*t*	*b*	*SE*	*t*	*b*	*SE*	*t*	*b*	*SE*	*t*
(Intercept)	.06	.08	.76	5.53	.02	**367**	5.58	.02	**334**	5.76	.02	**237**	5.71	.02	**241**
Preview															
Syntactically infelicitous alternative versus identical	−.15	.06	**−2.61**	.09	.02	**4.81**	.11	.02	**5.77**	.16	.03	**5.98**	.10	.02	**4.26**
Pseudocharacter versus syntactically infelicitous alternative	−.22	.06	**−3.88**	.01	.01	.99	.03	.02	1.74	.01	.02	.62	.03	.02	1.91
Frequency: High versus low	−.02	.05	−.35	.02	.01	1.30	.02	.01	1.62	.04	.02	**2.27**	.03	.02	**2.11**
Change awareness	−.66	.15	**−4.34**	.08	.03	**2.62**	.11	.03	**3.47**	.13	.05	**2.83**	.16	.05	**3.63**
Interactions															
Syntactically Infelicitous Alternative Versus Identical × Frequency	.51	.11	**4.47**	−.09	.03	**−2.99**	−.09	.03	**−3.04**	−.13	.04	**−3.10**	−.03	.03	−1.10
Pseudocharacter Versus Syntactically Infelicitous Alternative × Frequency	−.10	.11	−.87	.04	.03	1.34	.04	.03	1.42	.07	.04	1.85	.01	.03	.48
Syntactically Infelicitous Alternative Versus Identical × Change Awareness	−.18	.11	−1.59	.05	.03	1.41	.10	.04	**2.63**	.09	.05	1.73	.11	.04	**2.44**
Pseudocharacter Versus Syntactically Infelicitous Alternative × Change Awareness	−.13	.11	−1.14	−.00	.03	−.01	.01	.03	.29	.10	.04	**2.31**	.00	.03	.07
Frequency × Change Awareness	−.04	.09	−.44	−.03	.02	−1.34	−.01	.03	−.21	.03	.03	1.09	.00	.03	.15
Syntactically Infelicitous Alternative Versus Identical × Frequency × Change Awareness	.03	.23	.14	−.06	.06	−1.10	−.07	.06	−1.12	−.08	.09	−.96	−.06	.06	−.98
Pseudocharacter Versus Syntactically Infelicitous Alternative × Frequency × Change Awareness	−.03	.23	−.14	−.04	.06	−.61	−.06	.06	−.98	.03	.08	.33	−.06	.06	−1.08
*Note*. Significant terms are marked in bold, and marginally significant items are underlined. *b* = regression coefficient.															

## Footnotes

1 When the target word class was included in the analyses as a covariate, the same pattern of effects occurred, indicating that skipping effects hold for words in different syntactic categories (e.g., verbs and nouns). The syntactic category of the preview had no influence on skipping behavior.

2 To provide formal statistical support for the null interaction of task with preview and frequency, we undertook Bayes Factor analyses for linear mixed models (Morey et al., 2018) for skipping probability. Bayes Factors both for the model containing or excluding interactions of task with preview and frequency (i.e., *BF*_with interaction_, *BF*_without interaction_) were calculated. By comparing the two models (*BF* = *BF*_with interaction_/*BF*_without interaction_), we evaluated whether task interacted with preview and/or frequency. BF values smaller than 1 favor the null hypothesis, whereas *BF* values greater than 1 favor the alternative hypothesis. We used the default scale prior (*r* = 0.5) and 100,000 Monte Carlo iterations of the BayesFactor package, and the results of Bayesian analysis favored the null hypothesis (*BF* = 0.001). Also, a sensitivity analysis with different priors (i.e., 0.2, 0.3, 0.4, 0.5, 0.6, 0.7, and 0.8) provided consistent results (all *BF*s < 0.06).

3 When collapsing data from the two experiments, for the low-frequency target the effect was very robust with higher skipping rates when readers had a syntactically infelicitous alternative higher frequency preview compared to an identical (low-frequency) preview, *b* = 0.27, *SE* = 0.09, *z* = 2.86.

4 When display change awareness was included in the LMM analyses as a centered continuous predictor, the pattern of results was exactly the same to that reported using a categorical approach.

5 As in Footnote 2, similar Bayes factor analyses were conducted for skipping probability. We compared two models with or without interactions of change awareness with preview and frequency (*BF* = *BF*_with interaction_/*BF*_without interaction_), and the results of Bayesian analyses favored the null hypothesis (*BF* = 0.00003). Also, a sensitivity analysis with different priors (i.e., 0.2, 0.3, 0.4, 0.5, 0.6, 0.7, and 0.8) provided consistent results (all *BF*s < 0.01).
